# mSphere of Influence: Clearing a Path for High-Resolution Visualization of Host-Pathogen Interactions *In Vivo*

**DOI:** 10.1128/mSphere.00308-19

**Published:** 2019-07-10

**Authors:** Shumin Tan

**Affiliations:** a Department of Molecular Biology and Microbiology, Tufts University School of Medicine, Boston, Massachusetts, USA

**Keywords:** *Mycobacterium tuberculosis*, host-pathogen interactions, optical clearing

## Abstract

Shumin Tan works in the field of Mycobacterium tuberculosis-host interactions. In this mSphere of Influence article, she reflects on how the paper “Single-cell phenotyping within transparent intact tissue through whole-body clearing” by B. Yang et al. (Cell 158:945–958, 2014, https://doi.org/10.1016/j.cell.2014.07.017) impacted her ideas on approaches to visualize and understand heterogeneous host-pathogen interactions *in vivo* in 3-dimensional space at the single-cell level, through the tractable and broadly compatible tissue optical clearing methods developed.

## COMMENTARY

Inspiration for one’s work can come from subject areas that are quite different, with conceptual parallels or methodological advances crossing fields. I highlight here a paper—“Single-cell phenotyping within transparent intact tissue through whole-body clearing,” by the group of Viviana Gradinaru (1)—which describes a method with origins in neurobiology that I believe has significant potential for broad use in the field of infectious diseases. Their paper sought to develop methodology that would enable whole-organ imaging at the cellular level, while retaining complete intact tissue architecture and compatibility with myriad current imaging tools such as fluorescent proteins and RNA single-molecule fluorescence *in situ* hybridization (FISH) (1). In particular, their optimization of a passive tissue clearing method (PACT [for “passive clarity technique”]), together with development of a mounting medium compatible with use in imaging thick tissue (RIMS [for “refractive index matching solution”]), presents very tractable and easily applied methods that can be exploited in the study of host-pathogen interactions at the single-bacterium level, and in the spatial context of host tissue structure and function.

A main principle behind tissue optical clearing lies in the removal of packed lipid bilayers that impede both light and antibody penetration (2–4). There has been immense work in neurobiology in developing methods for optical clearing of brain tissue, driven by the need to understand structure-function relationships in the brain in the context of the intact organ (3–5). Yang et al. built on this foundation, utilizing a shared approach of tissue cross-linking and hydrogel hybridization to maintain structure integrity, with lipid extraction using ionic detergents (1). They optimized the cross-linking and hydrogel reagents and procedures, with a focus on compatibility with endogenous fluorophores and histological procedures (1). Different hydrogel formulations and detergents were tested, with 4% acrylamide and 8% sodium dodecyl sulfate ultimately found to be the best combination for balancing the different desired properties of (i) speed and robustness of optical clearance; (ii) maintenance of tissue architecture and protein epitopes for immunofluorescence analysis; and (iii) retention of the ability of macromolecules to penetrate the tissue, required for successful immunofluorescence and histological procedures (1). Importantly, they also developed a mounting medium, RIMS, that was straightforward and cost-effective to make and that allowed matching of the refractive index for optimal imaging (1). Their paper further presented a novel whole-body clearing method in mice that exploited perfusion of clearing reagents through the vasculature to enable optical clearing of all organs in a much shorter time period. Immunolabeling of all organs could also be done simultaneously with this PARS (perfusion-assisted agent release *in situ*) procedure, with both antibodies and small-molecule dyes delivered via the vasculature and with the washes required between the optical clearance and antibody administrations also exploiting the circulatory system (1).

The technological advances developed by Yang et al., particularly the passive optical clearing method of PACT and the use of RIMS, excitingly represent a method that is straightforward and easily applied to any organ and that, importantly, is compatible with fixed tissue and downstream immunofluorescence analysis without the need for highly specialized equipment. Particularly in the context of Mycobacterium tuberculosis-host interaction studies that my group is focused on, heterogeneity across multiple facets of the infection has been of burgeoning interest due to the consequences of this nonuniformity for infection outcome and treatment success. This heterogeneity ranges from lesion-level progression and outcome to variation in the local environment experienced by single bacilli even within a given lesion or host cell (6–14). Indeed, the concept of a critical role of heterogeneity in multiple host-pathogen interactions has been increasingly appreciated, with studies spanning Vibrio cholerae and *Salmonella* infection of the intestine to Yersinia pseudotuberculosis colonization of the spleen (15–17). However, understanding the structure-function relationships that underlie infection heterogeneity requires single bacterium-level analysis in intact tissue, which is inherently technically challenging. The use of optical clearing methods such as PACT, in combination with other imaging tools such as reporter bacterial strains, provides the opportunity to interrogate in 3-dimensional space differences in local bacterial responses during infection. It further provides a unique window into understanding how drug treatment or vaccination affects the bacteria at the single-bacterium level, in the context of host cell type, lesion structure, and local immune response.

The paper by Yang et al. has been extensively cited since its publication in 2014. There has been additional work on methods for optical clearing of tissue, as well as application of the clearing methodology to various fields, spanning visualization of blood brain barrier permeability in studies of prion disease (18) to the examination of histone methylation levels in intact xenograft tumors (19). In the field of infectious diseases, a few studies have begun to exploit the potential of optical clearing in analyzing host-pathogen interactions. PACT has been used in virus-host studies, with Kieffer et al. examining human immunodeficiency virus-1 spread, in concert with CD4^+^ T cell visualization, in lymphoid tissues in humanized mice (20). DePas et al. used PACT in combination with bacterial rRNA detection to examine pathogen aggregation patterns in sputum from cystic fibrosis patients (21). Optical clearing methods have also been used to examine the effect of macrophage epithelial reprogramming on granuloma cellular structure in a Mycobacterium marinum-zebrafish model (22), as well as the heterogeneity in granuloma tumor necrosis factor expression observed in this infection system (23). To examine local M. tuberculosis responses in intact lung tissue, we have started to combine PACT with our reporter M. tuberculosis strains that inform on the local environment experienced by the bacteria, or on bacterial replication status, in a C3HeB/FeJ murine model that exhibits a range of lesion types, including classic caseating granulomas (24). Finally, PACT has very recently been utilized to analyze Helicobacter pylori colonization establishment and persistence in the murine stomach (25). These studies have demonstrated the power of optical clearing methods such as PACT in revealing novel aspects of host-pathogen interactions, and it will be exciting to see the discoveries to come from its wider application.
